# Generalizability Theory in the Evaluation of Psychological Profile in Track and Field

**DOI:** 10.3390/sports12050127

**Published:** 2024-05-04

**Authors:** Cristina Sanz-Fernández, Verónica Morales-Sánchez, Julen Castellano, Antonio Hernández Mendo

**Affiliations:** 1 Department of Education Sciences, University of La Rioja, 26006 Logroño, Spain; 2 Department of Social Psychology, Social Work, Anthropology and East Asian Studies, University of Málaga, 29071 Málaga, Spain; mendo@uma.es (A.H.M.); vomorales@uma.es (V.M.-S.); 3 Departament of Physical Education and Sport, University of País Vasco, 01007 Vitoria-Gasteiz, Spain; julen.castellano@ehu.eus

**Keywords:** psychology, generalizability theory, variance components, psychological profile skills, track and field

## Abstract

Generalizability theory (GT) has been used throughout the scientific literature to ensure validity, reliability, and generalizability in different sport contexts. However, there is a small number of studies examining the measurement of psychological profiles in sport from this perspective. Therefore, this study’s main goal is the sources of variability and the optimal measurement design estimation for a good assessment of the psychological profile in track and field. The sample consisted of 470 participants (age: Average= 32.1; Standar Desviation = 13.5). The analysis of variance and generalizability component analysis has been performed in order to test the reliability and generalizability of the sample. The profile included the following variables: flow, motivation (from Self-Determination Theory and Achievement Goals), self-confidence, and psychological skills. Results confirm that the sample has a high degree of reliability and generalizability in all the tested models. So, a detailed study on the validity, reliability, and generalizability of samples and measures should be an inherent element in the practice of psychological counseling in sports.

## 1. Introduction

The progress of sport psychology has been associated with the development and publication of several instruments to measure mental characteristics [1]. In this sense, it is important to know the psychological profile in sport, which includes a set of mental characteristics and abilities that define the athlete [2] because a holistic understanding of the psychological processes underlying this profile makes it necessary to include in it the different constructs that may be related to sport performance [3]. Many variables have been included separately in research on the sport psychological profile (e.g., motivation, self-confidence), although the most productive studies are those that group constructs for measuring the psychological profile, highlighting the research conducted in Spanish using the *Inventario Psicológico de Ejecución Deportiva* (IPED; [4,5]), which allows us to distinguish between the strengths and weaknesses of an athlete’s profile. This instrument is divided into the following factors: self-confidence (e.g., I see myself more as a loser than a winner during competitions), negative coping control (e.g., I get angry and frustrated during competition), attentional control (e.g., I become distracted and lose my concentration during competition), visu-imaginative control (e.g., Before the competition, I imagine myself as a winner), and visu-imaginative control (e.g., Before the competition, I imagine myself as a winner and before the competition, I imagine myself executing my actions and performing perfectly), motivational level (e.g., I am highly motivated to do my best in the competition), positive coping control (e.g., I can maintain positive emotions during the competition), and attitudinal control (e.g., During the competition, I think positively).

Flow [6], self-determined motivation [7], achievement goals [8], and self-confidence [9], in addition to psychological skills, have been considered important variables that correspond to the psychological profile shown in this research. However, no studies have been found analyzing these variables in the field of athletics as a whole.

From this perspective, it is clear that any measurement tool should be analyzed to check its validity. Several ways of testing the validity of a measure have been described in the scientific literature. Nevertheless, the concept of validity has begun to be described as the degree of appropriateness of the inferences and interpretations that can be drawn from scores on measurement instruments [10]. In this way, evidence related to the purpose and use of a given instrument, including content, predictive, and construct evidence, is gathered and considered as evidence of the acceptable or unacceptable degree of validity of a test, depending on its use with a particular population [11]. As a result of research and reflection on the subject, generalizability theory (GT; [12,13]) emerged. GT is defined as a way of reducing the influence of sources of error, optimizing measurement designs and considering the reliability and generalizability of estimated measures. GT can be used to examine the sources of variation affecting a particular measurement. In addition, the use of GT makes it possible to estimate the fit of the measure to the mean of all possible observations [14]. In this way, all variance components contributing to the error of an estimate are identified and measured, and strategies are applied to reduce the influence of these sources of error on the measure [15,16]. The use of GT in sport psychology is widely justified, for example, for the aprioristic approach versus a posteriori application of one instrument [14]. In this sense, any of the presented works can be considered from an a priori point of view as an exploration of an insufficiently known or studied research domain and as a way to prepare for larger-scale research. It is also possible that sample sizes can be better adjusted to the social reality of research in sport and physical activity psychology.

In summary, GT unifies the concepts of reliability, validity, and precision of a given measure through four steps: (1) definition of the study facets; (2) analysis of variance of the scores obtained on the study facets; (3) calculation of the error components; and (4) optimization of the generalizability coefficients.

Despite the growth of GT in the scientific literature [11,12,13,14,15,16], no study has been found that tests the degree of accuracy in the generalization of the results of the questionnaires used, with the exception of IPED [4,5]. In view of the above, the main objective of this research is to estimate the sources of variability and to estimate the optimal measurement designs for an adequate evaluation of the psychological profile in athletics based on the measures of flow, motivation, self-confidence, and psychological skills.

## 2. Material and Methods

### 2.1. Participants

The sample consisted of 470 participants (266 men and 204 women). Of all, 241 answered the questionnaire online and 229 answered the printed version. All of them practiced athletics in different specialties. Specifically, 148 were runners (not federated) and 322 were federated athletes, of whom 95 were sprinters and/or hurdlers, 143 were middle-distance and/or long-distance runners, 81 were event specialists, and 9 were decathletes or heptathletes. Their age ranged from 14 to 70 years (AVG: 32.1; SD: 13.5). The ages of the participants were grouped into three categories: U18 (N = 73) for those under the age of 18, Senior (N = 200) for those aged between 18 and 35, and Master (N = 184) for those over 35.

### 2.2. Instruments

Participants completed a pretest questionnaire to collect relevant socio-demographic data (year of birth, gender, athletic specialties, total time of practice, time of practice in the specialties, weekly training hours, best time achieved in a competition and month and year when that time was achieved, participation in other sports, existence of training partners, and coach’s education in athletics). They also completed a battery of five questionnaires. The Flow Dispositional Scale-2 (FDS-2; [17]) includes 36 items that are divided into the following scales: challenge–ability balance (*α* = 0.66), action–attention fusion (*α* = 0.57), clear goals (*α* = 0.72), unambiguous feedback (*α* = 0.72), task focus (*α* = 0.66), feeling of control (*α* = 0.69), loss of self-consciousness (*α* = 0.64), distorted sense of time (*α* = 0.55) and autotelic experience (*α* = 0.73). The Sport Motivation Scale (SMS; [18]) consists in 28 items divided into 7 scales: Intrinsic Motivation to Get Stimulation (*α* = 0.76), Intrinsic Motivation to Get Things (*α* = 0.83), Intrinsic Motivation to Know (*α* = 0.80), Identified Regulation (*α* = 0.68), Introjected Regulation (*α* = 0.64), External Regulation (*α* = 0.74), and No Motivation (*α* = 0.75). The Task and Ego Orientation in Sport Questionnaire (TEOSQ; [19]) consists of 13 items and consists of 2 scales measuring task orientation (*α* = 0.78) and ego orientation (*α* = 0.80). The Self-Confidence in Sport Questionnaire (CACD; [20]) is composed of 7 items (*α* = 0.90) with a Likert-type response format from 1 to 7 (1 = completely disagree; 7 = completely agree). The Psychological Inventory of Sport Performance (IPED; Hernández-Mendo, 2006; Hernández-Mendo et al., 2014) consists of 42 items divided into the following factors: self-confidence (*α* = 0.77); negative coping control (*α* = 0.76); attentional control (*α* = 0.82); visuo-imaginative control (*α* = 0.81); motivational level (*α* = 0.75); positive coping control (*α* = 0.74); and attitudinal control (*α* = 0.76).

### 2.3. Procedure

Two questionnaire response formats were used for data collection: online and by hand. In both cases, anonymity and confidentiality of the information provided were ensured. First, an analysis of variance components was carried out, followed by an analysis of generalizability.

### 2.4. Data Analysis

Data analysis has been carried out using two main techniques: analysis of variance components and generalizability analysis. The following programs have been used: SAGT [21] and SAS System v.9.1 [22,23].

GT attempts to identify and measure the variance components contributing error to an estimate and, knowing them, implement strategies to reduce their influence on the measurement [24]. Reliability is estimated by being certain that the scale is measuring what it measures in a reproducible manner. The generalizability coefficient provides information on the stability and consistency of individual differences between people, as well as other possible sources of variation, by considering the fit of the models to the General Linear Model through the comparison of the residual variance of the least squares and maximum likelihood procedures. Different procedures can be distinguished to perform the analysis of variance components, although in this case, only two will be used, namely, least squares through the VARCOMP procedure and maximum likelihood through the GLM (Generalized Linear Model) procedure.

## 3. Results

### 3.1. Analysis of Variance Components

A variance component analysis of variance was performed using VARCOMP (method = type1) and GLM procedures for a six-facet model [y = p o a c f g], where (p): Participant; (o): Response Format; (a): Athlete Type; (c): Questionnaire; (f): Factor; and (g): Gender.

Due to the saturation produced by working with such a large number of facets, the model [y = p o a c f g] without interactions was used first (see Table 1). A similar error of variance was obtained with both procedures (GLM = 83,850.60/VARCOMP = 83,851), and the model and the facets [p], [c], [f] were significant (<0.001 <0.0001) and explained 95.30% of the variance. The rest of the facets collapsed because of the contribution of the [p] facet to the model.

Then, another analysis without interactions was performed with the model [y = o a c f g] to know the contribution of each facet, disregarding facet [p], where *(o): Response Format; (a): Athlete Type; (c): Questionnaire; (f): Factor; and (g): Gender.*

The model and all facets were significant. The model explained 91.88% of the variance. The error variance with both procedures was practically equal (GLM = 144,694.80/VARCOMP = 144,695), as can be seen in Table 2.

From this analysis, the four facets that contributed the most variance to the model were considered, and two new analyses were performed with all interactions with the model [y = a|c|f|g] and [y = a|c|f|o]. In the model [y = a|c|f|g], all facets with their interactions were significant, and 91.88% of the variance was explained, as can be seen in Table 3. The error variance with both procedures was very similar (GLM = 12,159/VARCOMP = 12,064).

On the other hand, for the model [y = a c f o], all facets with their interactions were significant, and 91.88% of the variance was explained. The error variance with both procedures was also very similar (GLM = 12,064/VARCOMP = 12,159). These results can be seen in Table 4.

With these estimated results on the equality in the error variance of both a least squares and a maximum likelihood procedure, it can be assumed that the sample is linear, normal, and homoscedastic [25,26].

### 3.2. Generalizability Analysis Results

From the models estimated in the analysis of variance components, the generalizability analysis was performed using the SAGT statistical program [21]. Eleven models with different designs were obtained: [f][a][c]/[o]; [f][a][c]/[o]; [o][a][c]/[f]; [o][a][f]/[c]; [o][f][c]/[a]; [g][a][f]/[c]; [g][a][f]/[c]; [a][c][f]/[g]; [g][c][f]/[a]; [g][a][c]/[f]; and, finally, [c][f]/[p]. The results obtained for all of them are described below.

When a generalizability analysis was performed with the different cross-facet designs on the different models in the model [f][a][c]/[o], where *(f) Factor; (a) Athlete Type; (c) Questionnaire;* and *(o) Response Format,* generalizability coefficients greater than 0.99 were obtained (relative G = 0.998 and absolute G = 0.998), confirming the generalizability of the numerical structure of the sample.

In the [f][a][c]/[o] model, the highest percentage of variance was associated with facet [a] (Athlete Type) with 39.020% followed by facet [c] (Questionnaire type) with 32.275% (Table 5). On the other hand, the lowest percentage of variance was associated with the interaction [o][f] (Questionnaire format and Factor, respectively) with 0.000%, followed by the interaction [a][f] (Athlete Type and Factor] with 0.013%.

In the model [o][a][c]/[f] (see Table 6), where *(o): Response Format; (a): Athlete Type; (c): Questionnaire;* and *(f): Factor,* generalizability coefficients greater than 0.98 were obtained (relative G = 0.998 and absolute G = 0.989). These results confirm that the factors estimated from the Response Format, the Type of Athlete, and the Questionnaire used present a high reliability and a high capacity for the generalization of the numerical structure with the sample studied.

In the [o][a][f]/[c] model, generalizability coefficients above 0.87 (relative G = 0.966 and absolute G = 0.872) are obtained, which confirms that the numerical structure of the sample studied has adequate reliability and generalizability.

In the [o][f][c]/[a] model, generalizability coefficients above 0.71 (relative G = 0.952 and absolute G = 0.713) are obtained, confirming that the number of athletes in the sample estimated from the remaining facets is reliable and generalizable.

In the model [g][a][f]/[c], where *(g): Gender; (a): Type of Athlete; (f): Factor;* and *(c): Questionnaire,* generalizability coefficients higher than 0.87 were obtained (relative G = 0.967 and absolute G = 0.872). These results show that the questionnaires estimated on the basis of Gender, Type of Athlete, and Factor present a high reliability and an adequate generalization capacity of the numerical structure of the sample studied.

In the [g][a][f]/[c] model, the highest percentage of variance was associated with facet [a] (Type of Athlete) with 39.020% followed by facet [c] (type of Questionnaire) with 32.275% (Table 7). The lowest percentages of variance were found in the interaction [g] [f] (Gender and Factor) with 0.000% of variance associated and the interaction between all facets of the model, which obtained 0.007%.

In the model [a][c][f]/[g] (see Table 6), where *(a): Athlete Type; (c): Questionnaire; (f): Factor;* and *(g): Gender,* optimal generalizability coefficients were obtained (relative G = 1.000 and absolute G = 1.000), confirming the reliability and optimal generalizability of the numerical structure of the studied sample. In the [g][c][f]/[a] model, generalizability coefficients higher than 0.71 (relative G = 0.950 and absolute G = 0.712) are obtained. These results confirm the reliability of the numerical structure of the sample studied and an adequate generalization capacity. In the model [g][a][c]/[f], generalizability coefficients above 0.98 (relative G = 0.998 and absolute G = 0.989) are obtained, showing the high reliability and generalizability of the numerical structure of the studied sample. And finally, the model [c][f]/[p], where *(c): Questionnaire; (f): Factor;* and *(p): Participant,* generalizability coefficients above 0.99 were obtained (relative G = 1.000 and absolute G = 0.995). These results show the generalizability of the numerical structure of the sample studied and a high reliability.

In the [c][f]/[p] model, the highest percentage of variance is associated with facet [p] (Participant) with 65.864% followed by facet [c] (Questionnaire type) with 29.000% (Table 8). However, the lowest associated variance percentages are found in facet [f] (Factor) and interaction [p][f] (Participant and Factor) with 0.000% and 0.361%, respectively.

## 4. Discussion

The general objectives of this research were to estimate the sources of variability and to calculate the optimal measurement designs for an adequate assessment of the psychological profile in athletics (consisting of flow, motivation, self-confidence, and psychological skills).

In order to study the facets that explain a greater percentage of the variance, variance component analysis and generalizability analysis were carried out. The aim was to ensure the generalizability of the data provided and the fit of the model, as well as to determine the variability of the facet involved [4]. With regard to the analysis of variance components, the results confirm that the main source of variance for the estimation of the psychological profile in athletics is the facet of Participants when the model is tested without interactions. This means that the variability that occurs between participants is large enough to determine their psychological profile. When this facet is disregarded, all the other facets and their interactions are significant. This means that the psychological profile in athletics is explained by the variability of the measured factors. For that reason, different explanatory models on the variance of the profile were tested. All of them explain 91.88% of the variance and show a very similar error variance, which allows us to assume that the sample is linear, normal, and homoscedastic [25,26]. These results also indicate that Gender, Questionnaire used, Type of Athlete, Response mode, and Factor are determinants in the study of the psychological profile and produce differences among participants.

With regard to the generalizability analysis, each of the proposed measurement designs was analyzed independently and reliability and generalizability indices were estimated with the aim of estimating the most parsimonious explanatory models that maximize the explained variance and minimize the error variance. All of them were adequate in terms of reliability and validity indices. Among all the models tested, the model [a] [c] [f]/[g], where [a] corresponds to the Type of Athlete, [c] to the Questionnaire used, [f] to the Factor, and [g] to the Gender of the participant, obtained optimal generalizability coefficients.

The use of generalizability theory in the measurement of the psychological profile of athletes, perhaps because of its novelty, is not widespread, but it has obtained positive results, e.g., [27]. Among other benefits, the use of generalizability theory in the study of psychological profiles allows the validity and reliability of the numerical structure used to be checked in a very precise way [16,28,29]. In addition, it makes it possible to estimate the sample, i.e., the minimum number of participants necessary to generalize the results [14]. In this sense, it can be affirmed that the approach of the present study has adequate reliability and generalizability indicators in the different models tested. The general analysis of the generalizability coefficients reveals that the generalization accuracy reliability of the results is optimal. Other studies have proved that GT is especially effective in the planification of investigations and interventions in sport [15,16]. These data contribute to considering that there are significant differences between the different athletic specialties, as well as between different genders or age groups. In this sense, it can be affirmed that the approach of this study has adequate reliability and generalizability indicators in the different models tested. The general analysis of the generalizability coefficients reveals that the reliability of the generalization accuracy of the results is optimal. These data contribute to considering that there are significant differences between the different athletic specialties, as well as between different genders or age groups.

It is effective to check that a research design is adequate in terms of the number of participants and/or observations and has the same precision of generalizability in subsequent analyses. But it has also helped us to be able to design a broader research design by introducing modifications to the current design through the use of nesting in the facets. This nesting can result in a higher generalization accuracy of an investigation. In fact, we will always have a smaller number of error components that will allow for such precision if the residuals are small. Indeed, in the spirit of Cronbach, a G-analysis is normally an a priori study, which serves to prepare a larger-scale research design [16]. The prior work of estimating the sources of variance should make it possible to fine-tune the measurement devices adapted to the decisions considered in the main investigation.

With this optimization plan in mind, future lines of research can be proposed to minimize the limitations of this study. This research has certain limitations that should be examined in future work. Among them, we can highlight the possibility of having carried out a previous analysis of the data regarding the role played by the questionnaire presentation format (online or on paper) and considering the interviewer’s bias in order to be able to carry out the analysis of the profile knowing the influence of this variable previously. This study could also have been approached by having collected other variables that would have been interesting for the analysis, such as sports level or experience in competitions.

However, these previous explorations, due to their rigor and precision, can serve as a basis for making decisions on a larger scale, which will help to confirm and extend the results obtained. A detailed study of the generalizability of the measure in the psychological profile within sport provides rigor to the studies that aim to explore it.

## Figures and Tables

**Table 1:** Analysis of variance of the model [y = p o a c f g].

Source	DF	Sum of Squares	Mean Square	F-Value	Pr > F
**Model**	501	1,699,027.56	3391.27	472.87	<0.0001
**Error**	11,692	83,850.58	7.17		
**Total corrected**	12,193	1,782,878.13			
	**R-squared**	**Coef Var**	**MSE root**	**and Media**	
	0.952969	24.79826	2.677988	10.79909	
**Source**	**DF**	**Type I SS**	**Mean Square**	**F-Value**	**Pr > F**
p	468	1,251,706.08	2674.59	372.94	<0.0001
o	0	0.00	-	-	-
g	0	0.00	-	-	-
a		0.00	0.00	0.00	1.00
c		441,834.84	55,229.36	7701.10	<0.0001
f		5486.64	261.27	36.43	<0.001
**Source**	**DF**	**Type III SS**	**Mean square**	**F-Value**	**Pr > F**
p	466	60,844.21	130.57	18.21	<0.0001
o	0	0.0000	-	-	-
g	0	0.0000	-	-	-
a		5.51	2.75	0.38	0.68
C		416,187.87	59,455.41	8290.37	<0.0001
F		5486.64	261.27	36.43	<0.0001

DF: degrees of freedom; Pr > F: probability greater than the calculated F; SS: Sum of Squares.

**Table 2:** Analysis of variance of the model [y = o a c f g].

Source	DF	Sum of Squares	Mean Square	F-Value	Pr > F
**Model**		1,638,183.34	46,805.24	3932.82	<0.0001
**Error**	12,158	144,694.79	11.90		
**Total corrected**	12,193	1,782,878.13			
	**R-squared**	**Coef Var**	**MSE root**	**and Media**	
	0.918842	31.94538	3.449812	10.79909	
**Source**	**Df**	**Type I SS**	**Mean Square**	**F-Value**	**Pr > F**
o	1	1407.84	1407.84	118.29	<0.0001
g	1	3007.19	3007.19	252.68	<0.0001
a		473,507.03	118,376.76	9946.62	<0.0001
c		668,180.33	83,522.54	7017.99	<0.0001
f		492,080.97	23,432.43	1968.91	<0.0001
**Source**	**DF**	**Type III SS**	**Mean Square**	**F-Value**	**Pr > F**
o	1	151.35	151.35	12.72	0.00
g	1	0.17	0.16	0.01	0.91
a		49,448.81	24,724.41	2077.47	<0.0001
c		414,453.02	59,207.57	4974.92	<0.0001
f		492,080.97	23,432.43	1968.91	<0.0001

DF: degrees of freedom; Pr > F: probability greater than the calculated F; SS: Sum of Squares.

**Table 3:** Analysis of variance of the model [y = a c f g].

Source	DF	Sum of Squares	Mean Square	F-Value	Pr > F
**Model**		1,638,031.99	48,177.41	4044.22	<0.0001
**Error**	12,159	144,846.14	11.91		
**Total corrected**	12,193	1,782,878.13			
	**R-squared**	**Coef Var**	**MSE root**	**Media**	
	0.918757	31.96077	3.451473	10.79909	
**Source**	**Df**	**Type I SS**	**Mean square**	**F-value**	**Pr > F**
g	1	3225.83	3225.83	270.79	<0.0001
a		474,468.74	118,617.19	9957.23	<0.0001
c		668,365.31	83,545.66	7013.18	<0.0001
f		491,972.11	23,427.24	1966.58	<0.0001
**Source**	**DF**	**Type III SS**	**Mean square**	**F-Value**	**Pr > F**
g	1	1.30	1.30	0.11	0.7410
a		49,427.83	24,713.91	2074.59	<0.0001
c		414,453.62	59,207.66	4970.14	<0.0001
f		491,972.10	23,427.24	1966.58	<0.0001

DF: degrees of freedom; Pr > F: probability greater than the calculated F; SS: Sum of Squares.

**Table 4:** Analysis of variance of the model [y = a c f o].

Source	DF	Sum of Squares	Mean Square	F-Value	Pr > F
**Model**		1,638,183.18	48,181.86	4048.82	<0.0001
**Error**	12,159	144,694.99	11.90		
**Total corrected**	12,193	1,782,878.13			
	**R-squared**	**Coef Var**	**MSE root**	**and Media**	
	0.918842	31.94408	3.449672	10.79909	
**Source**	**Df**	**Type I SS**	**Mean Square**	**F-Value**	**Pr > F**
o	1	1407.84	1407.84	118.30	<0.0001
a		475,395.05	118,848.76	9987.09	<0.0001
c		668,809.75	83,601.22	7025.17	<0.0001
f		492,570.52	23,455.74	1971.03	<0.0001
**Source**	**DF**	**Type III SS**	**Mean square**	**F-Value**	**Pr > F**
o	1	152.49	152.50	12.81	0.0003
a		49,458.12	24,729.06	2078.03	<0.0001
c		414,453.06	59,207.58	4975.33	<0.0001
f		492,570.52	23,455.74	1971.03	<0.0001

DF: degrees of freedom; Pr > F: probability greater than the calculated F; SS: Sum of Squares.

**Table 5:** Analysis of variance of the model [f][a][c]/[o].

F.V	S.C	G.L	C.M	Random	Mixed	Corr.	%	S.E
[o]	1407.84	1	1407.84	2.86	2.86	2.86	0.06	4.61
[a]	475,395.00	1	475,395.00	1778.66	1778.66	1778.66	39.02	1493.19
[o][a]	129.68	1	129.68	0.76	0.76	0.76	0.02	0.82
[c]	668,696.00		167,174.00	1471.22	1471.22	1471.22	32.28	930.79
[o][c]	2245.20		561.30	10.42	10.42	10.42	0.23	6.24
[a][c]	51,302.00		12,825.50	246.24	246.24	246.24	5.40	142.40
[o][a][c]	70.62		17.65	0.65	0.65	0.65	0.01	0.39
[f]	441,059.00		17,642.36	841.44	841.44	841.44	18.560	240.15
[o][f]	145.36		5.81	−0.95	−0.95	−0.95	0.00	0.41
[a][f]	577.71		23.11	0.62	0.62	0.62	0.01	0.73
[o][a][f]	342.20		13.69	2.57	2.57	2.57	0.06	0.75
[c][f]	80,409.00		804.09	199.59	199.59	199.59	4.38	28.15
[o][c][f]	248.23		2.48	0.83	0.83	0.83	0.02	0.18
[a][c][f]	409.06		4.09	1.64	1.64	1.64	0.04	0.29
[o][a][c][f]	82.10		0.82	0.82	0.82	0.82	0.02	0.12

Note. F.V = sources of variation; S.C = sum of squares; G.L = degrees of freedom; C.M = mean square; Aleato. = random; Correg = corrected; S.E. = standard error.

**Table 6:** Generalizability analysis of the different analyzed models.

Name of Securities	[f][a][c]/[o]	[o][a][c]/[f]	[o][a][f]/[c]	[o][f][c]/[a]	[g][a][f]/[c]	[a][c][f]/[a][c][f]/[g]	[g][c][f]/[a]	[g][a][c]/[f]	[c][f]/[p]
**O**	(2; INF)	(2; INF)	(2; INF)	(2; INF)	(2; 2)	(2; 2)	(2; 2)	(2; 2)	(470; INF)
**A**	(2; INF)	(2; INF)	(2; INF)	(2; INF)	(2; INF)	(2; INF)	(2; INF)	(2; INF)	(5; INF)
**C**	(5; INF)	(5; INF)	(5; INF)	(5; INF)	(5; INF)	(5; INF)	(5; INF)	(5; INF)	(26; INF)
**F**	(26; INF)	(26; INF)	(26; INF)	(26; INF)	(26; INF)	(26; INF)	(26; INF)	(26; INF)	(26; INF)
**Total observations**	520	520	520	520	520	520	520	520	520
**Relative G coefficient**	0.998	0.966	0.966	0.952	0.967	1.000	0.950	0.998	1.000
**Absolute G coefficient**	0.998	0.872	0.872	0.713	0.872	1.000	0.712	0.989	0.995
**Relative error**	8.029	92.037	92.037	126.648	90.708	0.000	132.305	8.034	0.002
**Absolute error**	9.460	386.280	386.280	1015.978	385.531	0.000	1020.006	40.348	0.046
**D. typical of relative error**	2.834	9.594	9.594	11.254	9.524	0.000	11.502	2.834	0.044
**D. standard absolute error**	3.076	19.654	19.654	31.874	19.635	0.000	31.938	6.352	0.213

INF: infinite.

**Table 7:** Analysis of variance of the model [g][a][f]/[c].

F.V	S.C	G.L	C.M	Random	Mixed	Corr.	%	S.E
[g]	3225.83	1	3225.83	8.43	8.43	4.21	0.09	10.54
[a]	474,469.00	1	474,469.00	1772.19	1775.40	1775.40	38.93	1490.29
[g][a]	913.01	1	913.01	6.42	6.42	6.42	0.14	5.74
[c]	667,789.00		166,947.25	1472.89	1474.12	1474.12	32.32	929.54
[g][c]	735.29		183.82	2.45	2.45	2.45	0.05	2.13
[a][c]	51,375.00		12,843.75	245.87	246.92	246.92	5.41	142.60
[g][a][c]	219.37		54.84	2.10	2.10	2.10	0.05	1.22
[f]	440,601.00		17,624.04	840.59	840.17	840.17	18.42	239.90
[g][f]	428.26		17.13	−0.84	−0.84	−0.84	0.00	0.80
[a][f]	630.05		25.20	−0.24	2.11	2.11	0.05	0.95
[g][a][f]	594.68		23.79	4.69	4.69	4.69	0.10	1.30
[c][f]	79,950.00		799.50	198.42	198.85	198.85	4.36	27.99
[g][c][f]	202.99		2.03	0.86	0.86	0.86	0.02	0.14
[a][c][f]	411.56		4.12	1.90	2.06	2.06	0.05	0.29
[g][a][c][f]	31.62		0.32	0.32	0.32	0.32	0.01	0.04

Note. F.V = sources of variation; S.C = sum of squares; G.L = degrees of freedom; C.M = mean square; Aleato. = random; Correg = corrected; S.E = standard error.

**Table 8:** Analysis of variance of the model [c][f]/[p].

F.V	S.C	G.L	C.M	Random	Mixed	Corr.	%	S.E
[p]	1,251,706.00	469	2668.88	20.47	20.47	20.47	65.86	1.34
[c]	441,832.00		110,458.00	9.01	9.01	9.01	29.00	5.22
[p][c]	13,686.00	1876	7.30	0.26	0.26	0.26	0.84	0.01
[f]	5043.85		201.75	−0.05	−0.05	−0.05	0.00	0.03
[p][f]	13,043.00	11725	1.11	0.11	0.11	0.11	0.36	0.00
[c][f]	31,703.00		317.03	0.67	0.67	0.67	2.17	0.09
[p][c][f]	25,864.00	46900	0.55	0.55	0.55	0.55	1.77	0.00

Note. F.V = sources of variation; S.C = sum of squares; G.L = degrees of freedom; C.M = mean square; Aleato. = random; Correg = corrected; S.E = standard error.

## Data Availability

The data presented in this study are available upon request from the corresponding author due to the privacy of the participants.
